# Death after Chloroform

**Published:** 1877-09

**Authors:** 


Death After Chloroform.—An inquest was recently held in Dawlish, Eng., touching the death of Sarah Crudge, æt. twenty-three years, who died suddenly soon after an operation for squint, and after chloroform had been administered. Deceased was thoroughly examined by Mr. Cann, the operator, and considered a fit subject for anæsthesia. Four drachms of chloroform were inhaled. After the operation deceased spoke to him and appeared perfectly right; she then fell into a sleep, her pulse being quiet and regular. In two and a half hours afterwards she was dead. The immediate cause of death was found to be effusion of blood on the brain.—*Med. Record.*

